# The Mediating Role of Insecure Attachment in the Gap in Parenthood Desire between Lesbian and Gay Individuals and Their Heterosexual Counterparts

**DOI:** 10.3390/ijerph20054084

**Published:** 2023-02-24

**Authors:** Geva Shenkman

**Affiliations:** Baruch Ivcher School of Psychology, Reichman University (IDC Herzliya), Herzliya 4610101, Israel; geva.shenkman@runi.ac.il; Tel.: +972-523-589210

**Keywords:** parenthood desire, attachment avoidance, attachment anxiety, lesbian women, gay men, Israel

## Abstract

Previous studies have shown that lesbian and gay (LG) individuals, in comparison to their heterosexual counterparts, tend to report lower levels of parenthood desire. While numerous variables have been suggested to explain this gap in parenthood aspirations, no study has investigated the mediating role of avoidant attachment in the association between sexual orientation and parenthood desire. For that purpose, a sample of 790 cisgender Israelis aged 18–49 years (*M* = 28.27, *SD* = 4.76) was recruited using convenience sampling. Among the participants, 345 self-reported as predominantly or exclusively lesbian or gay and 445 self-reported as exclusively heterosexual. Participants completed online questionnaires assessing their sociodemographic characteristics, parenthood desire, and avoidant and anxious attachment styles. Mediation analyses were performed using the PROCESS macro, and the results revealed that LG individuals reported lower parenthood desire, higher avoidant attachment, and higher anxious attachment compared to heterosexual individuals. Moreover, avoidant attachment had a significant mediation effect in the association between sexual orientation and parenthood desire. The findings suggest that LG individuals are more likely to report higher avoidant attachment due to possible rejection and discrimination from family members and peers, and this may be associated with lower parenthood desire. The results contribute to the growing body of research on family formation and parenthood aspirations among LG individuals, and specifically studies aimed at delineating the factors that contribute to the gap in parenthood aspirations between sexual minority individuals and their heterosexual counterparts.

## 1. Introduction

An increasing number of lesbian and gay (LG) individuals are becoming parents, due to advancements in fertility technologies and the enactment of progressive legislation [1,2]. As a result, parenthood desire in this population has received significant research attention [3,4,5,6]. Previous studies have explored the reasons why LG individuals may have lower aspirations for parenthood compared to their heterosexual counterparts [7,8], proposing societal and legal challenges, internalized negative attitudes towards LGBTQ individuals, and lower levels of societal pressure to become a parent [9,10,11]. The current study explored whether insecure attachment styles, and specifically avoidant attachment, might also contribute to the difference in parenthood aspirations between LG and heterosexual individuals.

### 1.1. Parenthood Desires among LG and Heterosexual Individuals

The desire to become a parent is a crucial aspect of parenthood [12]. For LG and heterosexual individuals, parenthood desire may be accompanied by other indicators of parenthood aspirations, such as parenthood intention (i.e., an explicit plan to become a parent) and the estimated likelihood of becoming a parent (i.e., taking into account societal and cultural factors, including discriminatory policies that restrict LGB individuals’ access to parenthood) [6,13,14]. Studies have shown that, compared to their heterosexual counterparts, LG individuals have a lower desire for parenthood [14,15,16,17]. Researchers have proposed that this difference may be due to the numerous barriers to parenthood that LG individuals face, including legal restrictions, restrictive policies by some reproductive agencies, experiences of discrimination, and financial burdens associated with fertility treatments and surrogacy [18,19,20]. Additionally, many LG individuals anticipate social stigma related to parenthood. In this vein, they tend to report higher levels of interpersonal vulnerability and traumatic outlook, which may hinder their parenthood desire [7,11,21,22,23]. It was also suggested that gay men are specifically more vulnerable to reporting lower parenthood desire in comparison to heterosexual or lesbian women, as their access to fertility possibilities is more limited [6,7]. Gay men may also face discriminatory social polices targeted specifically at them [11]. However, other factors, such as insecure attachment styles, may also play a role in explaining the gap in parenthood desire between LG and heterosexual individuals. This requires further investigation.

### 1.2. Attachment Style and Sexual Orientation

Attachment theory suggests that early experiences with primary caregivers shape patterns of behavior, emotions, and expectations in relationships, which influence a person’s emotional state and interactions [24]. Researchers typically categorize attachment as secure, anxious, or avoidant [25]. Secure attachment helps to maintain emotional stability, while anxious attachment is associated with emotion regulation difficulties and can lead to the development of distress-inducing coping mechanisms [26,27]. Individuals with high attachment anxiety tend to report a fear of rejection, which is usually managed using emotional dependence behaviors, such as clinging to an attachment figure [28]. Avoidant attachment is characterized by a lack of trust in others during times of stress and the adoption of self-reliance strategies [24,29].

While some studies have suggested that attachment styles are relatively stable over the lifetime and even have a genetic component [30], other studies have examined the potential for attachment styles to change in response to significant experiences [31]. With respect to sexual orientation, it has been suggested that negative parental reactions to a child’s coming out process may alter the child’s working models of attachment [32], potentially making it more likely that they will adopt an avoidant stance towards their environment [33,34]. Similarly, the challenges faced by many sexual minority individuals, including harassment and rejection, may lead to increased reliance on avoidant coping strategies (i.e., an avoidance of intimacy and interdependence), thereby negatively impacting their ability to form stable relationships [35].

To date, only a few studies have compared the attachment styles of heterosexual and LG individuals. Some studies have found no significant differences between these populations [36,37]. However, recent quantitative studies have found that LG individuals tend to exhibit higher levels of insecure attachment, and particularly avoidant attachment [16,38,39,40]. This may be explained by minority individuals’ tendency to experience discrimination and oppression, as well as rejection from parents and peers, which subsequently impact attachment schemes and behaviors [34,40].

### 1.3. The Mediating Role of Avoidant Attachment in the Gap in Parenthood Desire between LG and Heterosexual Individuals

As mentioned above, previous research has consistently detected lower levels of parenthood desire among LG individuals, in comparison to their heterosexual counterparts [15,17,41]. Studies have also detected higher levels of insecure attachment styles among LG, relative to heterosexual, individuals, particularly with regard to avoidant attachment [40]. Moreover, it has been shown that higher avoidant attachment, but not anxious attachment, is associated with lower parenthood desire [16,42]. A potential interpretation of this pattern is that individuals with avoidant attachment tend to show more reluctance to enter new relationships, and typically cope with their fear of rejection by avoiding new interactions, including couplehood and parenthood. In contrast, individuals with anxious attachment tend to use greater seeking behaviors to cope with their sensitivity to rejection, thereby establishing relationships to soothe their fears of abandonment; for this reason, their desire to be in a romantic relationship or a parent may be less impaired [42]. Despite these findings, the mediating role of avoidant attachment in the association between sexual orientation and parenthood desire has not yet been explored. This was the focus of the current study, and the findings represent an original contribution to the literature. 

Moreover, the current study aimed at extending previous mediation models proposing that differences in parenthood aspirations between LG and heterosexual individuals may be partly explained by LG individuals’ fear of being stigmatized as parents, interpersonal vulnerability, subjective traumatic stress, and social support [7,8,11,23]. The current study tested the role of a potential new mediator, avoidant attachment, in explaining the difference in parenthood aspirations between LG and heterosexual individuals. This variable is linked with previous social relationships and social support mediators explaining differences in parenthood aspirations between LG and heterosexual individuals, namely relationships with parents, romantic partners, and close friends [8]. The suggested attachment style differences may be an underlying mechanism of differences in perceptions of positive social relationships and social support, therefore fueling prior findings [8] while also offering a new angle by attachment theory. 

### 1.4. The Israeli Sociocultural Context

The current study drew on the theoretical perspective of family systems theory [43], which posits that an individual’s development is shaped by the larger societal and cultural context. The study took place in Israel, which offers a unique context for studying the different parenthood desire of LG versus heterosexual individuals, and the potential role of attachment avoidance in mediating this difference. 

Israeli society places a high value on family and parenthood [44,45], and it has one of the highest fertility rates among the Organization for Economic Co-operation and Development (OECD) countries [46]. However, Israeli laws and policies limit the ability of sexual minority individuals to become parents [2,47]. For instance, Israel offers very few adoption opportunities for LG individuals [48] and imposes bureaucratic hardships on those engaging in transnational surrogacy [49]. Furthermore, until January 2022, LG couples were prevented from accessing Israeli surrogacy services [50]. Such institutionalized discrimination may have a negative impact on the parenthood aspirations of Israeli sexual minority individuals [11].

### 1.5. Research Hypotheses

Based on the literature suggesting that LG individuals, compared to heterosexual individuals, tend to exhibit a lower desire for parenthood [14,15,16,17] and higher levels of insecure attachment [16,38,39,40], alongside findings showing that higher avoidant attachment, but not anxious attachment, is associated with lower parenthood desire [16,42], it was expected that (a) relative to their heterosexual counterparts, LG individuals would report lower levels of parenthood desire and higher levels of anxious and avoidant attachment; and (b) avoidant attachment, in turn, would be associated with lower parenthood desire.

Specifically, the following hypotheses were proposed:

**H1:**  *LG individuals would report lower parenthood desire, relative to heterosexual individuals;*

**H2:**  *LG individuals would report higher avoidant and anxious attachment styles, relative to heterosexual individuals;*

**H3:**  *Avoidant attachment would mediate the association between sexual orientation and parenthood desire, such that the predicted lower parenthood desire among LG individuals relative to their heterosexual counterparts would be accounted for by their greater avoidant attachment.*

## 2. Materials and Methods

### 2.1. Participants

Participants included 790 cisgender Israelis aged 18–49 years (*M* = 28.27, *SD* = 4.76). Of these, 43.7% (*n* = 345) self-reported as predominantly or exclusively lesbian or gay and 56.3% (*n* = 445) self-reported as exclusively heterosexual. With regard to gender, 52.9% identified as a woman and 47.1% as a man. Slightly less than half (45.4%) reported having a partner, and 61.1% had a college degree or higher. Most participants were Israeli by birth (93.9%), lived in a city (88.3%), self-reported as Jewish (97.1%), self-reported as secular (83.1%), and had a mean score of 3.31 (*SD* = 0.94) on their self-rated economic status, indicating average income on a 5-point scale ranging from 1 (*low economic status*) to 5 (*high economic status*).

Preliminary analyses were conducted to identify potential covariates by examining sexual orientation differences in the demographic variables, using chi-square tests (i.e., for gender, place of birth, relationship status, place of residence, education, family religion, and self-rated religiosity) and *t*-tests (i.e., for age and self-rated economic status). Table 1 presents the findings. More men self-reported as predominantly or exclusively gay than heterosexual. In contrast, more women self-reported as heterosexual than predominantly or exclusively lesbian. This difference in sexual orientation between men and women was significant. Compared to heterosexual participants, LG participants were significantly older, more likely to live in a city, less likely to have a college degree or higher, and less likely to be in a romantic relationship (see Table 1). No significant differences were found between LG and heterosexual participants with respect to place of birth, self-rated economic status, family religion, and self-rated religiosity. Table 1 presents the descriptive characteristics of the study groups and the coding categories for the variables.

### 2.2. Measures

*Demographics.* Sexual orientation was assessed using a 7-point self-rating scale [51] ranging from 0 (*exclusively heterosexual*) to 6 (*exclusively homosexual*). Individuals who self-reported as exclusively (i.e., 6 on the Kinsey scale) or predominantly (i.e., 5 on the Kinsey scale) homosexual were identified as LG. Heterosexual participants self-reported as exclusively heterosexual (i.e., 0 on the Kinsey scale). This method of confirming participants’ self-identification as exclusively or predominantly LG or heterosexual replicated the procedure applied in many previous studies in the field [52,53]. Self-report data were also collected for other sociodemographic factors, such as gender, place of birth, relationship status, place of residence, education, self-rated economic status, family religion, and self-rated religiosity. 

*Parenthood desire.* Parenthood desire was assessed using a single item: “If you are not a parent, please rank how strongly you want to become a parent.” Responses ranged from 1 (*not at all*) to 10 (*very much*). This question was adapted from Riskind and Patterson [13]. It has previously been used to assess parenthood desire [16,54], and has shown significant correlations with multiple scales of psychological well-being [54]. 

*Attachment orientation.* Avoidant and anxious attachment patterns were assessed using the Experiences in Close Relationships scale (ECR) [55]. The ECR is a 36-item self-report questionnaire that consists of two 18-item subscales: anxiety (e.g., “I worry about being abandoned”) and avoidance (e.g., “I don’t feel comfortable opening up to other people in close relationships”). Respondents are asked to think about their close relationships and rate the extent to which each item is self-descriptive on a 7-point scale ranging from 1 (*not at all*) to 7 (*very much*). The ECR was originally designed to assess experiences with recent romantic partners; however, researchers have broadened the ECR to assess “close relationships” [24]. In the present study, Cronbach’s alphas for avoidant and anxious attachment were 0.911 and 0.913, respectively. This instrument has been extensively used for research and clinical purposes worldwide [24], including among LG participants [40].

### 2.3. Procedure

Questionnaires were administered in Israel between November 2016 and March 2018. Participants were recruited via announcements posted on internet forums, social media, and an online newspaper. LG participants were recruited using targeted sampling, to ensure a sufficient number [56]. The announcements invited both sexual minority and heterosexual individuals to voluntarily and anonymously complete a survey on psychological coping with hardship. This survey had been used by the researchers for several studies, drawing on varied criteria. The announcements included a link to an online web survey (hosted on the Qualtrics online platform), and 1189 individuals clicked through to access the survey. The current study involved only childfree individuals under the age of 50 years (as older age is not commonly considered consistent with childbearing) [54,57]. All survey items that addressed parenthood desire, avoidant attachment, anxious attachment, self-identification as a woman or man, and self-identification as predominantly or exclusively lesbian/gay or exclusively heterosexual were mandatory in order to be included in the analytic sample. Therefore, individuals who were parents, aged 50 years or older, did not completely answer all mentioned items and scales, self-identified as neither a woman nor a man, and/or self-identified as neither lesbian, gay, nor heterosexual, were excluded from the analyses (total of 399). Of the 1189 who accessed the online survey, 66.44% met the abovementioned inclusion criteria, resulting in a total sample of 790. All participants were informed that their responses were anonymous and that participation was voluntary. All provided consent to participate. Participants were invited to write to the researchers if they had any questions, in order to ensure a thorough debriefing. Prior to data collection, the study was reviewed and approved by the Institutional Review Board of the Baruch Ivcher School of Psychology at Reichman University for compliance with standards for the ethical treatment of human participants (ethical clearance number: P_2017007).

### 2.4. Data Analysis

Data analysis was conducted using SPSS 28 (IBM, New York, NY, USA). The preliminary analyses involved chi-square tests and *t*-tests examining sexual orientation differences in the demographic variables, aimed at identifying potential covariates. To investigate the study hypotheses and further examine whether avoidant and anxious attachment mediated the association between sexual orientation and parenthood desire, the PROCESS macro (model 4) [58] was implemented (controlling for gender, age, education, relationship status, and place of residence, which significantly differed between groups). This macro assesses the significance of the cross-product of the coefficients of the predictor of the mediator relation (path A) and the mediator of the outcome relation, while controlling for the predictor (path B). To test the significance of the mediation effect, Hayes’s [58] method was applied using 5000 bootstrapped samples in order to estimate the 95% bias-corrected and accelerated confidence intervals (CIs) of the indirect effect of sexual orientation on parenthood desire through avoidant and anxious attachment, respectively. A significant mediation effect was confirmed when the CI of the indirect effect did not include zero. The AB cross-product test is considered the gold standard for exploring mediation or indirect effects, and it is typically recommended over more traditional mediation approaches [58,59,60]. As it was more specifically aimed at exploring the mediating role of avoidant attachment in the association between sexual orientation and parenthood desire, two separate mediation analyses were conducted (the first with avoidant attachment and the second with anxious attachment as a mediator). Conditional process analyses (i.e., moderated mediation) were then conducted, in an exploratory perspective, to identify potential differentiation in the mediation models as a function of gender. Therefore, to explore the possible moderating effect of gender in the association between sexual orientation and avoidant attachment (i.e., path A), model 7 was conducted. To explore the possible moderation of gender in the association between avoidant attachment and parenthood desire (path B), model 14 was conducted. Finally, and also in an exploratory perspective, four group analyses using one-way ANCOVAs were conducted (with gay men, heterosexual men, lesbian women, and heterosexual women), to explore possible group differences in parenthood desire, avoidant attachment, and anxious attachment. 

## 3. Results

As shown in Table 2, higher levels of attachment avoidance were correlated with lower parenthood desire. In addition, higher attachment avoidance was correlated with higher attachment anxiety.

**Figure 1:**
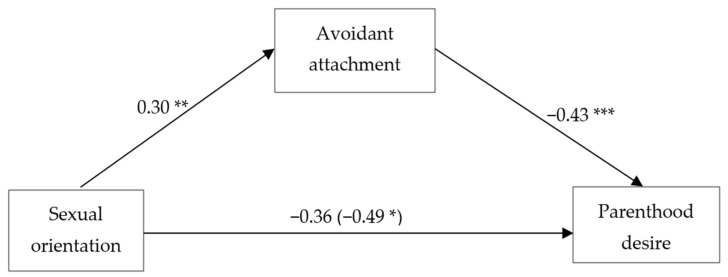
The mediation effect of avoidant attachment on the association between sexual orientation and parenthood desire. Note. *N* = 789. The reported values are unstandardized regression coefficients (*B*s) for the pathways between sexual orientation (0 = heterosexual, 1 = sexual minority), avoidant attachment, and parenthood desire. Gender, age, education, relationship status, and place of residence were used as covariates. The total effect of sexual orientation on parenthood desire is reported in parentheses. * *p* < 0.05. ** *p* < 0.01. *** *p* < 0.001.

Mediation analyses were conducted (controlling for gender, age, education, relationship status, and place of residence) to test whether LG individuals had lower parenthood desire than heterosexual individuals, whether LG individuals had a greater prevalence of avoidant and anxious attachment styles relative to heterosexual individuals, and whether avoidant attachment mediated the disparity in parenthood desire between LG and heterosexual individuals. The results (see Figure 1) indicated that LG individuals tended to report lower parenthood desire. Moreover, LG individuals showed a greater prevalence of avoidant attachment, which was subsequently associated with lower parenthood desire. The CIs of the indirect effect did not contain zero (mediated *b* = −0.13, *SE* = 0.04, 95% CI [−0.23, −0.06], Sobel *Z* = −2.86, *SE* = 0.05, *p* = 0.004). Thus, avoidant attachment had a significant mediation effect on the association between sexual orientation and lower parenthood desire. As shown in Figure 1, avoidant attachment fully mediated the association between sexual orientation and parenthood desire, as the direct effect of sexual orientation on parenthood desire (*B* = −0.49, *p* = 0.018) decreased and became non-significant (*B* = −0.36, *p* = 0.077) when it was mediated by avoidant attachment. Notably, the mediation effect remained significant even when anxious attachment was added to the model as a controlled variable, as the CIs of the indirect effect did not contain zero (mediated *b* = −0.10, *SE* = 0.04, 95% CI [−0.19, −0.03], Sobel *Z* = −2.43, *SE* = 0.04, *p* = 0.015). 

**Figure 2:**
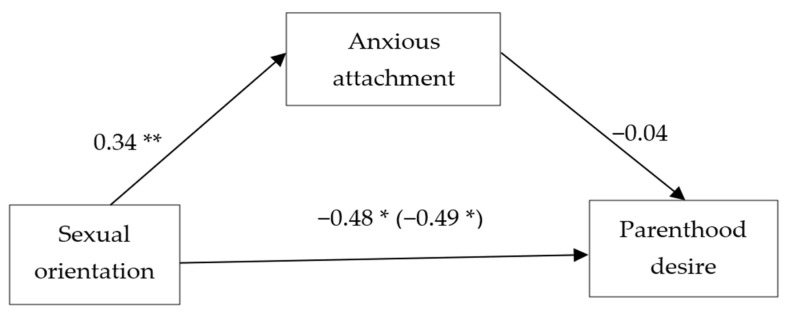
The mediation effect of anxious attachment on the association between sexual orientation and parenthood desire. Note. *N* = 789. The reported values are unstandardized regression coefficients (*B*s) for the pathways between sexual orientation (0 = heterosexual, 1 = sexual minority), avoidant attachment, and parenthood desire. Gender, age, education, relationship status, and place of residence were used as covariates. The total effect of sexual orientation on parenthood desire is reported in parentheses. * *p* < 0.05. ** *p* < 0.01.

A similar analysis was conducted to examine the mediation effect of anxious attachment on the association between sexual orientation and parenthood desire. As shown in Figure 2, LG individuals tended to report lower parenthood desire. While LG individuals showed a greater prevalence of anxious attachment, this attachment style was not associated with lower parenthood desire. The CI of the indirect effect did contain zero (mediated *b* = −0.01, *SE* = 0.03, 95% CI [−0.08, 0.04], Sobel *Z* = −0.42, *SE* = 0.03, *p* = 0.674). Thus, anxious attachment did not have a significant mediation effect on the association between sexual orientation and parenthood desire. Notably, the same pattern of result remained when avoidant attachment was also added to the model as a controlled variable, as the CIs of the indirect effect did contain zero (mediated *b* = 0.01, *SE* = 0.02, 95% CI [−0.03, 0.07], Sobel *Z* = 0.56, *SE* = 0.02, *p* = 0.576).

To explore whether the significant mediation model of avoidant attachment in the association between sexual orientation and parenthood desire differed as a function of gender (i.e., men vs. women), conditional process analysis was applied, focused on the possible moderating effect of gender in the association between sexual orientation and avoidant attachment. The Cis of the moderated mediation indices [58] did not contain zero (index = −0.13, *SE* = 0.07, 95% CI [−0.29, −0.07]) when predicting parenthood desire using avoidant attachment as a mediator, and the conditional indirect effect of the mediation model was significant for women (mediated *b* = −0.20, *SE* = 0.06, 95% CI [−0.33, −0.09]), but not men (*b* = −0.06, *SE* = 0.05, 95% CI [−0.16, 0.03]). Hence, the mediation of avoidant attachment in the association between sexual orientation (LG vs. heterosexual individuals) and parenthood desire was significantly moderated by gender (in path a), showing a significant mediation model for women but not for men. This pattern was also confirmed when running the mediation models separately for lesbian and heterosexual women (model 4; total effect = −0.15, *p* = 0.596; direct effect = 0.06, *p* = 0.838; indirect effect = −0.21, *SE* = 0.08, 95% CI [−0.39, −0.09], Sobel *Z* = −2.68, *SE* = 0.08, *p* = 0.007) and for gay and heterosexual men (model 4; total effect = −0.86, *p* = 0.005; direct effect = −0.79, *p* = 0.009; indirect effect = −0.06, *SE* = 0.05, 95% CI [−0.19, 0.01], Sobel *Z* = −1.21, *SE* = 0.05, *p* = 0.224). 

A similar moderated mediation analysis focusing on the possible moderation of gender in the association between avoidant attachment and parenthood desire was also conducted. The Cis of the indices of moderated mediation [58] contained zero (index = 0.00, *SE* = 0.06, 95% CI [−0.12, 0.13]). Hence, the reported indirect effect was unrelated to gender.

Four group analyses were also conducted with gay men (*n* = 228), heterosexual men (*n* = 144), lesbian women (*n* = 117), and heterosexual women (*n* = 301), using one-way ANCOVAs to explore group differences in parenthood desire, avoidant attachment, and anxious attachment. The results indicated that parenthood desire differed across the four groups (*F* (3, 786) = 5.952, *p* < 0.001, η_p_^2^ = 0.022). Pairwise comparisons, using Bonferroni-corrected post hoc tests, revealed that gay men (*M* = 6.91, *SD* = 2.92) scored significantly lower on parenthood desire than heterosexual men (*M* = 7.95, *SD* = 2.29; *p* < 0.001), heterosexual women (*M* = 8.20, *SD* = 2.27; *p* < 0.001), and lesbian women (*M* = 7.85, *SD* = 2.76; *p* = 0.007). All other pairwise companions were insignificant.

The results also showed that avoidant attachment differed across the four groups (*F* (3, 786) = 7.362, *p* < 0.001, η_p_^2^ = 0.028). Pairwise comparisons, using Bonferroni-corrected post hoc tests, revealed that gay men (*M* = 3.33, *SD* = 1.09) scored significantly higher on avoidant attachment than heterosexual women (*M* = 2.88, *SD* = 1.00; *p < 0*.001), and lesbian women (*M* = 3.36, *SD* = 1.10) scored significantly higher on avoidant attachment than heterosexual women (*M* = 2.88, *SD* = 1.00; *p* < 0.001). Heterosexual women (*M* = 2.88, *SD* = 1.00) scored significantly lower on avoidant attachment than heterosexual men (*M* = 3.16, *SD* = 0.90; *p* = 0.045). All other pairwise comparisons were insignificant. 

Finally, anxious attachment also differed across the four groups (*F* (3, 786) = 6.510, *p* < 0.001, η_p_^2^ = 0.024). Pairwise comparisons, using Bonferroni-corrected post hoc tests, revealed that gay men (*M* = 3.44, *SD* = 1.20) scored significantly higher on anxious attachment than heterosexual men (*M* = 2.97, *SD* = 1.16; *p* < 0.001), and lesbian women (*M* = 3.52, *SD* = 1.13) scored significantly higher on anxious attachment than heterosexual men (*M* = 2.97, *SD* = 1.16; *p* < 0.001). Heterosexual women (*M* = 3.31, *SD* = 1.10) scored significantly higher on anxious attachment than heterosexual men (*M* = 2.97, *SD* = 1.16; *p* = 0.023). All other pairwise companions were insignificant. 

## 4. Discussion

The current study aimed at extending previous mediation models explaining the difference in parenthood aspirations between LG and heterosexual individuals. Specifically, the study proposed avoidant attachment as a mediator in the association between sexual orientation and parenthood desire. Consistent with the first hypothesis, LG individuals reported lower parenthood desire than their heterosexual counterparts. In line with the second hypothesis, LG individuals reported higher levels of avoidant and anxious attachment styles in comparison to their heterosexual counterparts. In line with the third hypothesis, avoidant attachment had a significant mediation effect in the association between sexual orientation and parenthood desire. 

The finding that LG individuals reported lower parenthood desire in comparison to heterosexual individuals is aligned with prior research [3,15,17,40,41,61]. This gap in parenthood desire between LG and heterosexual individuals may be explained by the legal and bureaucratic difficulties faced by many LG individuals, the financial burden associated with the use of assisted reproductive technologies and cross-border surrogacy, and LG individuals’ anticipation of being stigmatized as parents [11,18,20,62]. Moreover, internalized homonegativity among LG individuals and the greater pressure placed on heterosexual couples to have children may also contribute to the gap in parenthood desire between LG and heterosexual individuals [9,10].

In the current study, gay men reported the lowest level of parenthood desire. This may be understood in light of gay men’s limited routes to parenthood in the Israeli context. For example, in Israel, both heterosexual couples and lesbian women are eligible for Health Maintenance Organization (HMO) coverage for assisted reproductive procedures, most likely due to the pronatalist and familistic culture [44,45]. However, gay men in Israel have far fewer opportunities to become parents and receive HMO support. For instance, prior to 2022, only heterosexual couples and single women were allowed to access surrogacy services within Israel, and gay couples and single men were excluded [50]. This discriminatory policy may have heightened gay men’s awareness of the difficulties associated with the parenthood journey, possibly hindering their parenthood aspirations [63]. Other characteristics of Israeli society (i.e., the patriarchal culture, which promotes masculine stereotypes; and the orthodox Jewish religion, which tends to condemn and stigmatize gay men) may also explain the increased stigma and discrimination experienced by gay men, which may also limit their parenthood aspirations [2].

In line with the second hypothesis, LG individuals reported higher levels of both avoidant and anxious attachment styles, in comparison to their heterosexual counterparts. These results are aligned with recent findings suggesting more insecure attachment styles among LG than heterosexual individuals [16,38,40]. These findings could be interpreted in light of the possible associations between insecure attachment styles and both sexual minority identity and experiences of discrimination, harassment, and minority stress [32,34]. It has been suggested that common hurdles faced by LG individuals due to their sexual minority status, such as rejection from parents and siblings, harassment from peers, and the internalization of homonegative attitudes, may lead to increased rejection sensitivity, which could manifest in a greater avoidance of intimacy or a more anxious-ambivalent pattern of attachment, thereby impacting the security of their relationships [32,35,40]. 

In line with prior findings [42], the current study found that higher avoidant attachment, but not higher anxious attachment, was associated with lower parenthood desire. This pattern could be explained by the fact that individuals with avoidant attachment tend to avoid new relationships and interactions, including parenthood, as a response to their fear of rejection in relationships. On the other hand, individuals with anxious attachment tend to seek out relationships, sometimes compulsively, to alleviate their fear of abandonment; this coping mechanism does not necessarily impede their desire to be in a romantic relationship or become a parent [42]. 

Moreover, the current findings—notably the mediation effect of avoidant attachment in the association between sexual orientation and parenthood desire—point to the emerging role of avoidant attachment as a variable explaining the gap in parenthood aspirations between LG and heterosexual individuals. The current results suggest that LG individuals are more prone to report avoidant attachment, which in turn is associated with lower parenthood desire. This finding makes a direct contribution to current mediation models explaining the gap in parenthood aspirations between LG and heterosexual individuals, which rely on variables such as anticipation of stigma upon parenthood, interpersonal vulnerability, and subjective traumatic outlook [11,23,62]. Furthermore, this finding aligns with previous mediational frameworks suggesting that the social support context, i.e., relationship with parents, romantic partners, and close friends, mediate the association between sexual orientation and parenthood aspirations [8]. It could be suggested that attachment theory may be another framework to use to understand the mechanisms of social relationships and social support and their impact on parenthood desire among sexual minority individuals. The current finding also contributes to the growing scientific literature aimed at identifying the variables that contribute to parenthood aspiration [7,8,63]. The investigation of such mediating variables is especially pertinent in the Israeli pronatalist and familistic context, which associates parenthood with social acceptance and greater life meaning [64,65].

Intriguingly, the current findings specified that the mediation of avoidant attachment in the association between sexual orientation and parenthood desire was moderated by gender, with significance for women but not for men. This identifies the specific vulnerability of lesbian women compared to heterosexual women, with regard to the gap in parenthood desire accounted for by avoidant attachment. This pattern echoes previous findings utilizing social support variables as mediators in the association between sexual orientation and parenthood aspirations between LG and heterosexual individuals, and in which mediation models also worked better for lesbian women than for gay men [8]. This adds convergent validity and strengthens the argument that attachment style differences may underlie differences in perceptions of social support and contexts when studying gaps in parenthood desire between LG and heterosexual individuals. The moderation of gender in the mediation of avoidant attachment in the association between sexual orientation and parenthood desire may be understood in light of the Israeli study context. In Israel’s pronatalist culture, motherhood is an essential identity characteristic and frequently considered a “national mission” [66]. Therefore, Israeli women may be subjected to significant pressure to become mothers, as well as socialization processes accentuating familism [67]. Lesbian women, who are prone to dual discrimination (i.e., discrimination based on both gender and sexual orientation), seem more likely than heterosexual women to report high avoidant attachment, which in turn appears to be associated with decreased parenthood desire. In addition, Israel’s patriarchal, machoistic culture [68] may contribute to an atmosphere in which women—and especially lesbian women—face more stigma, harassment, and discrimination [69,70]. Nevertheless, this effect of gender in the suggested mediational model requires further investigation, and future studies are called to explore this effect more deeply. 

### Strengths and Limitations

The current study explored the mediating role of avoidant attachment in the association between sexual orientation and parenthood desire. The findings provide important insight into the differing levels of parenthood desire between LG and heterosexual individuals. However, several limitations should be noted. First, as the study relied solely on self-report measures, the data may have been affected by self-presentation biases. Second, the groups were not based on random or representative samples. Third, as the research design was correlational, causal inferences could not be drawn. Fourth, most participants were living in an urban environment, educated to a university level, and secular, and had an average or high economic status; thus, the sample was fairly homogenous, and not necessarily representative of all Israeli LG people (thereby limiting the generalizability of the results). Fifth, the measure of parenthood desire consisted of only one item, which may pose validity and reliability limitations. Sixth, while the unique study context of Israel represents a strength of the study, it also imposes cultural restrictions that, again, limit the generalizability of the results. 

Future studies should explore the suggested mediation model in other social contexts, and among other sexual minorities, such as bisexual and transgender individuals. Such exploration is needed among diverse sexual minorities in light of stereotypes and prejudice that LGBTQ+ individuals are still facing in modern societies [71,72]. Additionally, building on recent studies comparing LG and heterosexual parents on their desire for more children [73], future studies should explore the potential mediation of attachment in the association between sexual orientation and the desire for more children in these groups. As the majority of previous research on parenthood desire and attachment styles among sexual minorities is quantitative, it is also recommended that future qualitative studies will explore participants’ experiences in connection with this research topic, to deepen the understanding of the association between attachment style, parenthood aspirations, and sexual orientation. Many of the abovementioned methodological limitations reflect common difficulties of research with sexual minority populations [74].

## 5. Conclusions

The current study found lower levels of parenthood desire, alongside higher avoidant and anxious attachment styles, among LG individuals, relative to heterosexual individuals. Furthermore, avoidant attachment was found to mediate the association between sexual orientation and lower parenthood desire.

The findings suggest that, despite improvements in fertility technologies and a more liberal sociopolitical climate relating to sexual minority rights, LG individuals still tend to report lower parenthood desire in comparison to heterosexual individuals. Moreover, LG individuals are more likely to report an insecure attachment orientation (i.e., avoidant and anxious), and avoidant attachment is associated with lower parenthood desire. Reproductive healthcare professionals, counsellors, and mental health professionals should be mindful of these associations between attachment style and parenthood aspirations. Policymakers should also be aware of the possible links between sexual orientation, attachment avoidance, and lower parenthood desire, possibly influenced by the broader atmosphere of policies that discriminate against LG individuals [50]. Moreover, social agents and policymakers should work to promote equal rights and a safe environment for sexual minority individuals. Finally, researchers should further explore the role of variables such as familism, pronatalism, and family support in the model proposed in the current study. 

To summarize, the current study proposed avoidant attachment as a mediating variable in the association between sexual orientation and parenthood desire. The findings contribute to the growing literature delineating factors that shape parenthood aspirations among sexual minority individuals.

## Figures and Tables

**Table 1:** Sociodemographic characteristics of the study groups.

Variable	Lesbian Women and Gay Men (*n* = 345)	Heterosexual Women and Men (*n* = 445)	Difference Test *t*/*χ*^2^	*p*
Gender % (*n*)			*χ*^2^ (1) = 88.73	*p* < 0.001
1. Men	66.1 (228)	32.4 (144)		
2. Women	33.9 (117)	67.6 (301)		
Age (range)	18–49	18–42	*t* (788) = −8.69	*p* < 0.001
*M*	*29.97*	*26.95*		
*SD*	*5.86*	*3.11*		
Place of birth % (*n*)			*χ*^2^ (1) = 1.42	*p* = 0.234
1. Israel	95.1 (328)	93.0 (414)		
2. Elsewhere	4.9 (17)	7.0 (31)		
Education % (*n*)			*χ*^2^ (1) = 9.34	*p* = 0.002
1. Academic degree	55.1 (190)	65.8 (292)		
2. No academic degree	44.9 (155)	34.2 (152)		
Couplehood % (*n*)			*χ*^2^ (1) = 5.02	*p* = 0.025
1. Not in relationship	59.1 (204)	51.1 (227)		
2. In relationship	40.9 (141)	48.9 (217)		
Place of residence % (*n*)			*χ*^2^ (1) = 7.48	*p* = 0.006
1. Rural	8.1 (28)	14.4 (28)		
2. Urban	91.9 (317)	85.6 (380)		
Self-rated economic status % (*n*)			*t* (787) = 0.749	*p* = 0.454
1. Low	5.5 (19)	3.2 (14)		
2. Below average	12.2 (42)	11.5 (51)		
3. Average	42.0 (145)	42.8 (190)		
4. Above average	29.6 (102)	34.5 (153)		
5. High	10.7 (37)	8.1 (36)		
*M*	*3.28*	*3.33*		
*SD*	*0.99*	*0.90*		
Family religion % (*n*)			*χ*^2^ (1) = 0.21	*p* = 0.648
1. Not Jewish	2.6 (9)	3.2 (9)		
2. Jewish	97.4 (336)	96.8 (429)		
Self-rated religiosity % (*n*)			*χ*^2^ (1) = 0.86	*p* = 0.353
1. Secular	81.7 (282)	84.2 (374)		
2. Traditional/orthodox	18.3 (63)	15.8 (70)		

Note: The *t*-tests for age and economic status compared the respective mean ratings of the compared groups.

**Table 2:** Pearson correlations between the main study variables.

Variable	*M*	*SD*	1	2	3
1. Parenthood desire	7.73	2.60	__	−0.20 ***	−0.03
2. Avoidant attachment	3.13	1.05		__	0.24 ***
3. Anxious attachment	3.32	1.16			__

Note. *N* = 790; *** *p* < 0.001.

## Data Availability

The study data are available upon request from the author.
